# Compromised neuroplasticity in cigarette smokers under nicotine withdrawal is restituted by the nicotinic α_4_β_2_-receptor partial agonist varenicline

**DOI:** 10.1038/s41598-017-01428-6

**Published:** 2017-05-03

**Authors:** G. Batsikadze, W. Paulus, A. Hasan, J. Grundey, M.-F. Kuo, M. A. Nitsche

**Affiliations:** 1 Department of Clinical Neurophysiology, Georg-August-University of Göttingen, Robert Koch Straße 40, 37075 Göttingen, Germany; 2 Department of Neurology, Essen University Hospital, University of Duisburg-Essen, Hufelandstraße 55, 45147 Essen, Germany; 3 Department of Psychology and Neurosciences, Leibniz Research Centre for Working Environment and Human Factors, Ardeystraße 67, 44139 Dortmund, Germany; 4 Department of Neurology, University Medical Hospital Bergmannsheil, Bürkle de la Camp-Platz 1, 44789 Bochum, Germany; 5 Department of Psychiatry and Psychotherapy, Ludwig Maximilian University, Nußbaumstraße 7, 80336 Munich, Germany

## Abstract

Nicotine modulates neuroplasticity and improves cognitive functions in animals and humans. In the brain of smoking individuals, calcium-dependent plasticity induced by non-invasive brain stimulation methods such as transcranial direct current stimulation (tDCS) and paired associative stimulation (PAS) is impaired by nicotine withdrawal, but partially re-established after nicotine re-administration. In order to investigate the underlying mechanism further, we tested the impact of the α_4_β_2_-nicotinic receptor partial agonist varenicline on focal and non-focal plasticity in smokers during nicotine withdrawal, induced by PAS and tDCS, respectively. We administered low (0.3 mg) and high (1.0 mg) single doses of varenicline or placebo medication before stimulation over the left motor cortex of 20 healthy smokers under nicotine withdrawal. Motor cortex excitability was monitored by single-pulse transcranial magnetic stimulation-induced motor evoked potential amplitudes for 36 hours after plasticity induction. Stimulation-induced plasticity was absent under placebo medication, whereas it was present in all conditions under high dose. Low dose restituted only tDCS-induced non-focal plasticity, producing no significant impact on focal plasticity. High dose varenicline also prolonged inhibitory plasticity. These results are comparable to the impact of nicotine on withdrawal-related impaired plasticity in smokers and suggest that α_4_β_2_ nicotinic receptors are relevantly involved in plasticity deficits and restitution in smokers.

## Introduction

Nicotine is the main component of tobacco responsible for the addictive properties of smoking. On the other hand, it positively impacts cognitive functions, such as working, episodic memory and attention^1–3^ in humans and animals. Clinical studies in patients with schizophrenia and Alzheimer’s disease have also revealed improvement of cognitive functions by nicotine^4^. However, other studies demonstrate negative or no effects of nicotine on cognition^2, 5^, which might partially be explained by baseline performance differences between participant groups^6^. The physiological foundation for these cognitive effects is hypothesized to be nicotinic modulation of intracellular Ca^2+^ concentration through α_4_β_2_ and α_7_ nicotinic acetylcholine receptors (nAChRs)^7^. These receptors are ligand-gated ion channels, involved in plasticity induction and cortical excitability modulation^8, 9^. In animal studies, with regard to plasticity, yet α_4_β_2_ and α_7_ nAChR activation yielded mixed results, producing enhancement of either LTP or LTD^8, 10^. Experiments in humans have shown that both, nicotine-induced non-selective activation of nAChRs, and targeted α_4_β_2_ and α_7_ receptor activation by respective agonists result in an increase of focal plasticity, while abolishing the effects of more diffuse plasticity induction protocols in non-smokers^11, 12^. In contrast, smokers under nicotine withdrawal display deficient facilitatory plasticity, probably caused by hypo-activation of desensitized nAChRs. In accordance, nicotine re-administration restitutes this impaired facilitatory plasticity^13^. The impact of nicotinic agents on glutamatergic plasticity is suggested to be mediated by nAChR-dependent neuronal calcium influx in non-smoking subjects. In accordance, administration of nicotine, and hereby enhancing calcium influx, reestablished LTP-like plasticity abolished by dextromethorphan, which blocks NMDA receptors with calcium channel properties^14^.

Plasticity in the above-mentioned studies was induced by non-invasive brain stimulation protocols, such as transcranial direct current stimulation (tDCS) and paired associative stimulation (PAS). Both techniques induce long-lasting Ca^2+^- and NMDA receptor-dependent shifts in cortical excitability^15, 16^. tDCS non-selectively affects neuronal populations under the relatively large stimulation electrodes via subthreshold resting membrane potential modulation, inducing non-focal plasticity^16, 17^, whereas PAS induces relatively focal and synapse-specific neuroplastic changes, affecting mainly synapses between motor and somatosensory neurons^15^. For tDCS, LTP-like plasticity is induced by anodal, and LTD-like plasticity by cathodal stimulation of the target area^16^, while for PAS synchrony of activation of somatosensory-motor cortical connections determines plasticity direction, resembling spike-timing dependent plasticity^15^.

Apart from disturbed facilitatory plasticity, numerous studies in humans have reported that abstention from nicotine in smokers leads to deficits in working memory and attention^18, 19^, which are partially restituted by re-administration of nicotine or a α_4_β_2_ nAChR agonists^20–22^. However, in smoking humans the role of specific nAChRs in the re-establishment of impaired plasticity has not yet been explored. The fact that the above-mentioned α_4_β_2_ nAChRs have calcium channel properties^7^ suggests their key role in the restitutive effect of nicotine on withdrawal-related impaired plasticity. Moreover, in non-smoking individuals, acute nicotine and varenicline (partial agonist to the α_4_β_2_ and full agonist to α_7_ nAChRs) administration produces fairly similar effects on stimulation-induced plasticity^12, 23^. Here, we aimed at exploring the effect of the α_4_β_2_ activation on impaired plasticity in smokers during nicotine abstinence. For this reason, we selected varenicline, due to its 4000–5000-fold greater affinity for the α_4_β_2_ compared to that for α_7_ nAChRs^24^. We expected that 0.3 mg and 1 mg dosages of varenicline should produce results similar to those of global nAChR activation and thus ameliorate nicotine withdrawal-related plasticity deficits in smokers.

## Materials and Methods

### Subjects

Twenty-six healthy smokers aged 24.5 ± 3.7 years (16 females/10 males) were recruited. All of them were students (BA, MSc and Ph.D.) of the University of Göttingen and naïve to the stimulation techniques. Six participants (4 females/2 males) from the initial group left the study after two or three experimental sessions: one participant (from the PAS experiment) cancelled the participation due to side effects and five participants (four from the tDCS, one from the PAS experiment) left the study due to their busy lesson schedules; data from these sessions were excluded from the analysis. From the remaining group of twenty participants, four (1 female/3 males) took part in both, the tDCS and PAS parts of the study, therefore a group of twelve subjects aged 25.4 ± 3.8 years (7 females/5 males) completed the tDCS and a group of twelve subjects aged 24 ± 3.4 years (6 females/6 males) completed the PAS experiment. No preliminary tests were conducted to check the responsiveness of the subjects to either tDCS or PAS. All subjects were right-handed according to the Edinburgh handedness inventory^25^. None of them took any medication, had a history of a neuropsychiatric or medical disease, present pregnancy, or metallic head implants. All subjects gave written informed consent and were compensated for participation. All subjects were smokers with a cigarette consumption of minimum 10 cigarettes a day for at least 5 years continuously and a Fagerström score of at least 1 point, indicating a light degree of nicotine dependence^26^. They were not allowed to smoke for 10 hours (3 to 4 half-lives of nicotine^27^) before and during the experimental sessions. The investigation was approved by the Ethics Committee of the University of Göttingen, and conforms to the principles laid down in the Declaration of Helsinki.

### Transcranial Direct Current Stimulation

tDCS was administered by a battery-driven constant current stimulator (neuroConn GmbH, Ilmenau, Germany) through a pair of rubber electrodes (with the cable connector centered on the side of the rubber pad) covered with saline-soaked sponges (35 cm^2^, 5 × 7 cm). The motor cortex electrode was fixed over the area representing the right abductor digiti minimi muscle (ADM) and the return electrode above the contralateral supraorbital area. Subjects received 1 mA of either excitability-enhancing anodal tDCS for 13 minutes or excitability-diminishing cathodal tDCS for 9 minutes over the primary motor cortex, which induces motor cortex excitability alterations lasting for about 1 h after intervention^28, 29^.

### Paired Associative Stimulation

The peripheral electric pulse over the right ulnar nerve at the level of the wrist at an intensity of 300% of the sensory perceptual threshold was delivered by a Digitimer D184 multipulse stimulator (Digitimer, Welwyn Garden City, United Kingdom) and was followed by a TMS pulse over the M1 representation of the abductor digiti minimi muscle (ADM) conducted by a Magstim 200 stimulator with an intensity to elicit single pulse MEPs of ~1 mV peak-to-peak amplitudes. In total, 90 paired pulses were delivered at a frequency of 0.05 Hz at ISIs of 10 ms (inhibitory PAS or PAS10) or 25 ms (facilitatory PAS or PAS25). During PAS, the participants were instructed to silently count the number of pulses to guarantee sufficient attention to the procedure, which has been shown to be crucial to obtain the desired after-effects^30, 31^.

### Pharmacological Interventions

Low (0.3 mg) or high (1.0 mg) dosages of varenicline or 0.5 mg placebo were administered in two-piece non-transparent gelatin capsules (size 2.18 mm length, 6.35 mm external diameter) three hours before the start of the non-invasive brain stimulation protocol, allowing the verum drug to reach its maximum plasma level^32^. 1 mg varenicline is a usual single oral dosage administered in smokers twice per day to support cessation of cigarette consumption^32–35^ and both 0.3 and 1 mg doses of varenicline had a modulatory effect on both tDCS- and PAS-induced plasticity in a previous study of our group^12^.

### Monitoring of motor cortical excitability

In order to measure excitability changes, MEPs were recorded from the right ADM by single-pulse TMS over the corresponding left primary motor cortex, conducted by a Magstim 200 magnetic stimulator (Magstim, Whiteland, Dyfed, United Kingdom) with a figure-of-eight magnetic coil (diameter of one winding −70 mm; peak magnetic field −2.2T). The coil was held tangentially to the skull, with the handle pointing posterior and laterally at 45° from the midline. The hotspot was defined as the coil placement, where the TMS pulse resulted consistently in the largest MEPs of the right ADM. Surface MEPs were recorded with Ag-AgCl electrodes in a belly-tendon montage. The signals were amplified, band-pass filtered (2 Hz to 2 kHz, sampling rate, 5 kHz) and digitized with a micro 1401 AD converter (Cambridge Electronic Design, Cambridge, UK), controlled by Signal Software (Cambridge Electronic Design, v. 2.13), and stored into a laboratory computer for offline analysis.

### Experimental procedures

**Figure 1:**
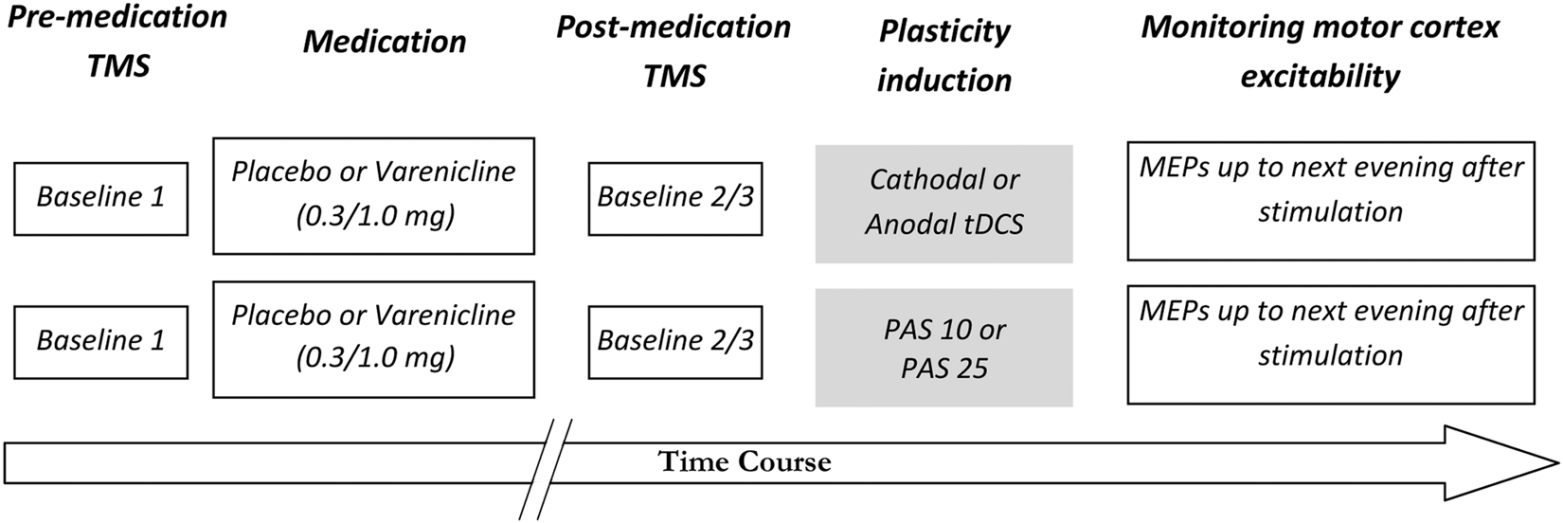
Course of the study. Participants were not allowed to smoke 10 h before and during the experimental session. In the beginning of each session, 25 baseline single pulse MEPs were recorded at an intensity to elicit MEPs with peak-to-peak amplitudes of on average ~1 mV before administration of varenicline (0.3 or 1 mg) or placebo medication. Three hours later, a second baseline was recorded to explore the effect of medication on cortical excitability, and the stimulation intensity was adjusted, if necessary (third baseline). Next, tDCS (cathodal or anodal) or PAS (PAS10 or PAS25) was administered and blocks of 25 MEPs were recorded at time points of 0, 5, 10, 15, 20, 25, 30, 60, 90 and 120 minutes after plasticity induction. Further TMS measurements were conducted in the evening of the same day (SE), morning (at ~9:00 AM, NM), noon (at ~12:00 PM, NN) and evening (at ~6:00 PM, NE) of the second day.

The participants were seated in a comfortable chair with head and arm rests. In the beginning, the motor cortex hotspot was identified by TMS and then the stimulation intensity was adjusted to elicit single pulse MEPs with peak-to-peak amplitudes of on average 1 mV. Then twenty-five MEPs were recorded for the determination of first baseline. To keep the EMG electrodes and TMS coil position constant throughout the session, their exact positions were marked with a waterproof pen. After first baseline recording, varenicline or placebo medication was administered. Three hours after intake of medication, a second baseline was recorded to monitor for a possible impact of the drug alone on cortical excitability (baseline 2), and TMS intensity was adjusted, if necessary (baseline 3). After determination of the second or third baseline, one of the plasticity induction protocols was applied (cathodal tDCS, anodal tDCS, PAS10 or PAS25) and twenty-five MEPs were recorded at time points of 0, 5, 10, 15, 20, 25, 30, 60, 90 and 120 minutes after tDCS. Further TMS measurements were conducted in the evening of the stimulation day (SE), next morning, at ~9:00 AM (NM), next noon, at ~12:00 PM (NN) and next evening, at ~6:00 PM (NE) (Fig. 1). To avoid interferences, the interval between two consecutive experimental sessions for a single subject was minimum seven days. Subjects were blinded for both, stimulation and medication conditions; the experimenter was blinded only for the medication condition.

### Analysis and statistics

The individual means of 25 MEP amplitudes were calculated at each time point for every subject. MEPs in which the muscle was not relaxed (i.e. observable EMG background activity present before the onset of the MEP) were excluded from the analysis.

#### Baselines

Individual baseline MEP amplitudes and maximum stimulator output (%MSO) percentage values for each session were analyzed with repeated measures analysis of variance (ANOVA) separately for tDCS and PAS experiments, using Baseline MEP or %MSO as dependent variable, respectively and session and medication (0.3 mg, 1.0 mg or placebo) as within-subject factors.

#### After-effects

post-stimulation mean MEP amplitudes were normalized to the respective mean baseline MEP amplitudes (quotient of post-stimulation vs pre-stimulation MEPs values: baseline 2, or, if TMS intensity was adjusted, baseline 3). Then the grand averages for each time point were calculated. A repeated measures ANOVA was performed on the above-mentioned data separately for tDCS and PAS experiments, using MEP amplitude as the dependent variable and medication, stimulation type and time course as within-subject factors.

The Mauchley’s test was used to evaluate the sphericity assumption and degrees of freedom were corrected using the Greenhouse-Geisser method when necessary. In case of significant results of the ANOVA, exploratory post hoc comparisons were performed using Student’s t-tests (paired samples, two-tailed, p < 0.05, not corrected for multiple comparisons) between the MEP amplitudes before and after intervention within one experimental condition and between the single time points (medication vs placebo) within the same stimulation condition.

Bonferroni-corrected t-tests were not used in the exploratory secondary analysis, because the experiment was powered for the primary statistical test, i.e. the ANOVA. Furthermore, since post hoc t-tests were also applied in the majority of our foregoing related studies^11–14, 23^, we used identical tests in order to improve inter-study comparability.

To compare main effects of different dosages of varenicline on plasticity, averaged MEPs for the first 30 minutes after stimulation were calculated for each subject per experimental session and normalized to baseline 2 (or 3, if TMS intensity was adjusted). These averaged MEP values for each dosage condition were tested via one-way ANOVA, exploratory post-hoc comparisons were conducted using Student’s t-tests (paired samples, two-tailed, p < 0.05, not corrected for multiple comparisons).

EMG measures were analyzed using custom python scripts based on the Stimfit library (version 0.14; available open source http://www.stimfit.org/doc/sphinx/)^36^. Statistical analysis was carried out with SPSS (version 23.0, IBM Corp.).

## Results

All subjects tolerated the procedure well. Only one subject (from the PAS experiment) experienced dizziness, nausea, and vomiting under placebo medication and left the study.

On average, the interval between two consecutive experimental sessions was 11.8 ± 9.07 days.

Results of the Fagerström scores were 3.0 ± 1.8 (min 1; max 6) for the PAS and 3.5 ± 1.4 (min 1; max 6) for the tDCS group, indicating mild-to-moderate nicotine dependence^37^, with no significant differences as revealed by one-way-ANOVA (F(1, 22) = 0.559, p = 0.462) between stimulation groups.

Due to time constraints of participants, data were missing for the second day measurements (NM, NN and NE time points) for three subjects; in total, 8 time points in the tDCS and 3 in the PAS experiment (0.74% and 0.27% of the data, respectively). The expectation maximization (EM) imputation method was used to replace the missing values^38, 39^.

### Effect of varenicline on motor cortex excitability

Varenicline and placebo alone did not have any impact on cortical excitability at any dosage, as revealed by the repeated measures ANOVAs conducted on the baseline data (independently on MEP amplitudes and %MSO) separately for tDCS and PAS experiments (for details see Table 1).

**Table 1:** MEP amplitudes and stimulation intensity before and after varenicline administration.

Stimulation	TMS Parameter	Medication condition	Baseline 1	Baseline 2	Baseline 3
Cathodal tDCS	MEP	0.3 mg	0.97 ± 0.13	1.01 ± 0.19	0.93 ± 0.13
		1.0 mg	0.94 ± 0.11	0.81 ± 0.13	0.92 ± 0.06
		Placebo	0.97 ± 0.13	1.06 ± 0.45	0.97 ± 0.12
	%MSO	0.3 mg	57.17 ± 14.48		57.08 ± 14.57
		1.0 mg	56.08 ± 14.15		56.67 ± 14.06
		Placebo	56.50 ± 13.57		56.75 ± 14.07
Anodal tDCS	MEP	0.3 mg	0.95 ± 0.08	0.95 ± 0.13	1.00 ± 0.11
		1.0 mg	0.99 ± 0.16	0.88 ± 0.15	0.93 ± 0.13
		Placebo	1.00 ± 0.13	0.97 ± 0.15	0.99 ± 0.13
	%MSO	0.3 mg	56.83 ± 13.11		57.25 ± 13.07
		1.0 mg	57.08 ± 14.37		57.83 ± 14.83
		Placebo	56.75 ± 14.00		57.17 ± 13.82
PAS10	MEP	0.3 mg	0.94 ± 0.10	1.05 ± 0.40	1.04 ± 0.15
		1.0 mg	0.99 ± 0.10	0.86 ± 0.12	0.90 ± 0.08
		Placebo	1.01 ± 0.14	0.93 ± 0.18	0.97 ± 0.10
	%MSO	0.3 mg	57.08 ± 11.22		57.17 ± 11.63
		1.0 mg	57.75 ± 11.95		58.25 ± 11.93
		Placebo	56.67 ± 12.35		57.08 ± 12.38
PAS25	MEP	0.3 mg	0.92 ± 0.11	0.91 ± 0.21	0.95 ± 0.12
		1.0 mg	0.97 ± 0.10	0.97 ± 0.19	0.98 ± 0.14
		Placebo	1.01 ± 0.10	0.89 ± 0.15	0.94 ± 0.11
	%MSO	0.3 mg	56.83 ± 12.34		57.00 ± 12.45
		1.0 mg	57.17 ± 11.85		57.58 ± 11.95
		Placebo	57.25 ± 11.42		57.58 ± 11.42

Shown are the mean MEP amplitudes ± S.D. and stimulation intensity (percentage of maximum stimulator output, %MSO) means ± S.D. of baselines 1, 2 and 3. The intensity of TMS was adjusted to elicit MEPs with peak-to-peak amplitude of ~1 mV (baseline 1). A second baseline (baseline 2) was recorded three hours after varenicline or placebo intake to determine the impact of the drug on cortical excitability and adjusted if necessary (baseline 3). RM-ANOVAs revealed no significant differences between %MSO values and MEP amplitudes.

### Effect of varenicline on tDCS-induced plasticity

The RM-ANOVA revealed a significant MEDICATION × STIMULATION × TIME (F(28) = 1.877; p = 0.006) interaction (for details see Table 2).

**Table 2:** Results of the repeated measures ANOVA.

Experiment	Factor	Df	F	p
tDCS	Medication	2	0.531	0.596
	Stimulation	1	11.862	0.005*
	Time	14	0.769	0.701
	Medication × stimulation	2	11.765	<0.001*
	Medication × time	28	0.518	0.981
	Stimulation × time	14	6.013	<0.001*
	Medication × stimulation × time	28	1.877	0.006*
PAS	Medication	2	0.486	0.621
	Stimulation	1	27.095	<0.001*
	Time	14	0.907	0.553
	Medication × stimulation	2	6.718	0.005*
	Medication × time	28	0.868	0.661
	Stimulation × time	14	2.897	0.001*
	Medication × stimulation × time	28	1.136	0.294

*Significant results at p < 0.05.

**Figure 2:**
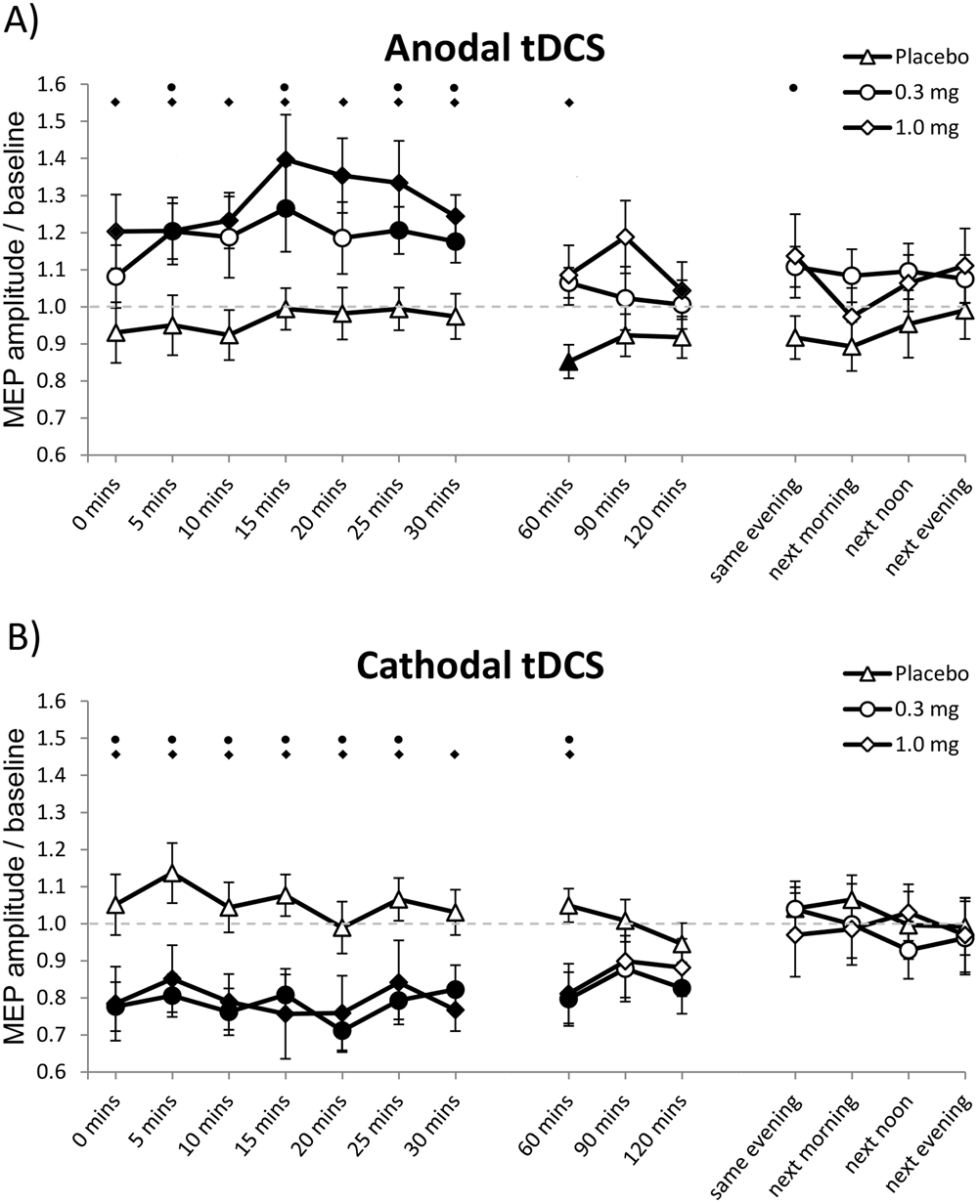
Impact of varenicline on tDCS-induced neuroplasticity. Shown are baseline-normalized MEP amplitudes after plasticity induction by anodal (**A**) and cathodal (**B**) tDCS under 0.3 mg, 1.0 mg varenicline or placebo medication conditions up to the evening of the post-stimulation day. (**A**) In smokers under placebo medication, anodal tDCS induced no excitability enhancement, while 0.3 mg and 1.0 mg varenicline resulted in enhanced MEP amplitudes after anodal tDCS. (**B**) In the placebo condition, cathodal tDCS failed to induce excitability alterations. In contrast, both low and high doses of varenicline led to significant inhibitory after-effects of tDCS. Error bars indicate S.E.M. Filled symbols indicate significant differences of post-stimulation MEP amplitudes from respective baseline values; Floating symbols indicate significant differences between the respective drug and placebo medication conditions at the same time points (Student’s t-test, two tailed, paired samples, p < 0.05).

Post-hoc Student’s t tests show that under placebo medication, tDCS induced no plasticity, as MEPs did not differ significantly from baseline values (the only exception being anodal tDCS at minute 60, where MEP size was significantly reduced compared to the baseline). Under both doses of varenicline, tDCS induced relevant excitability alterations. Cathodal tDCS induced a significant excitability diminution, lasting for 60 minutes after stimulation, whereas anodal tDCS induced an excitability enhancement, which was more prominent and stable under high dose varenicline (Fig. 2A,B).

**Figure 3:**
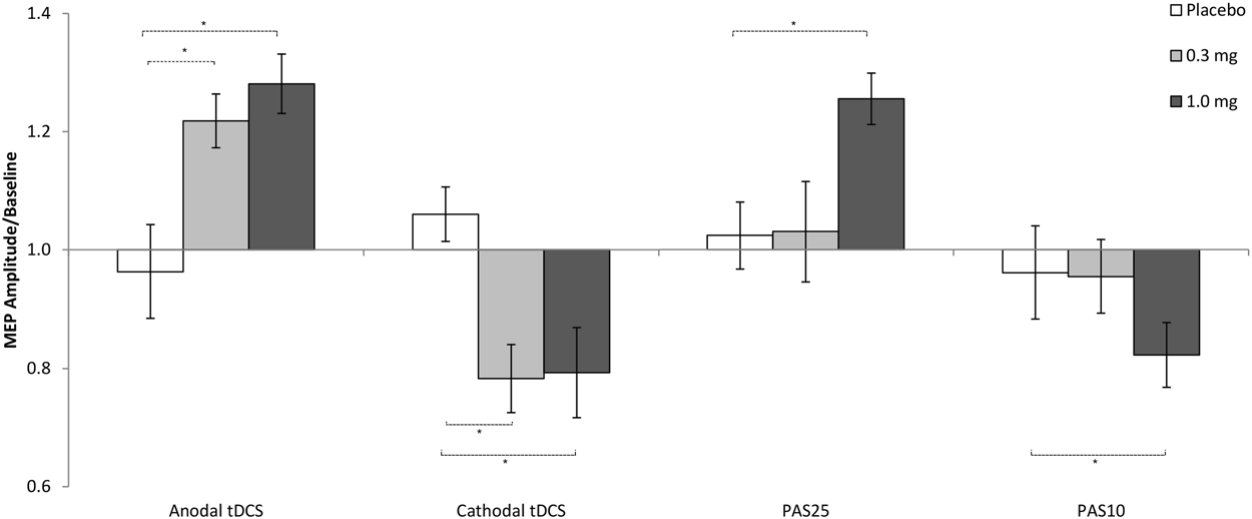
In smokers under placebo medication, tDCS- and PAS-induced plasticity is abolished. Furthermore, PAS also induced no excitability alterations under 0.3 mg varenicline. High dose varenicline restituted both tDCS and PAS-induced after-effects, while 0.3 mg varenicline only restored tDCS-induced plasticity. Each column represents the mean of baseline-normalized MEP ± S.E.M. amplitudes until 30 minutes after stimulation; Asterisks indicate significant differences between drug and placebo conditions (Student’s t-test, two tailed, paired samples, p < 0.05).

For the effects of different dosages of varenicline on tDCS-induced plasticity, the grand average values calculated for the first 30 min after intervention were compared across groups by a one-way ANOVA and were significantly different (F(5, 66) = 11.951, p < 0.001). Both excitability-enhancing and -diminishing after-effects were present under low and high doses of varenicline compared to placebo (Student’s t test, paired samples, two-tailed, p < 0.01), under which no after-effects were observed (Fig. 3).

### Effect of varenicline on PAS-induced plasticity

The RM-ANOVA revealed significant STIMULATION × TIME (F(14) = 2.897; p = 0.001) and MEDICATION × STIMULATION (F(2) = 6.718; p = 0.005) interactions (Table 2).

**Figure 4:**
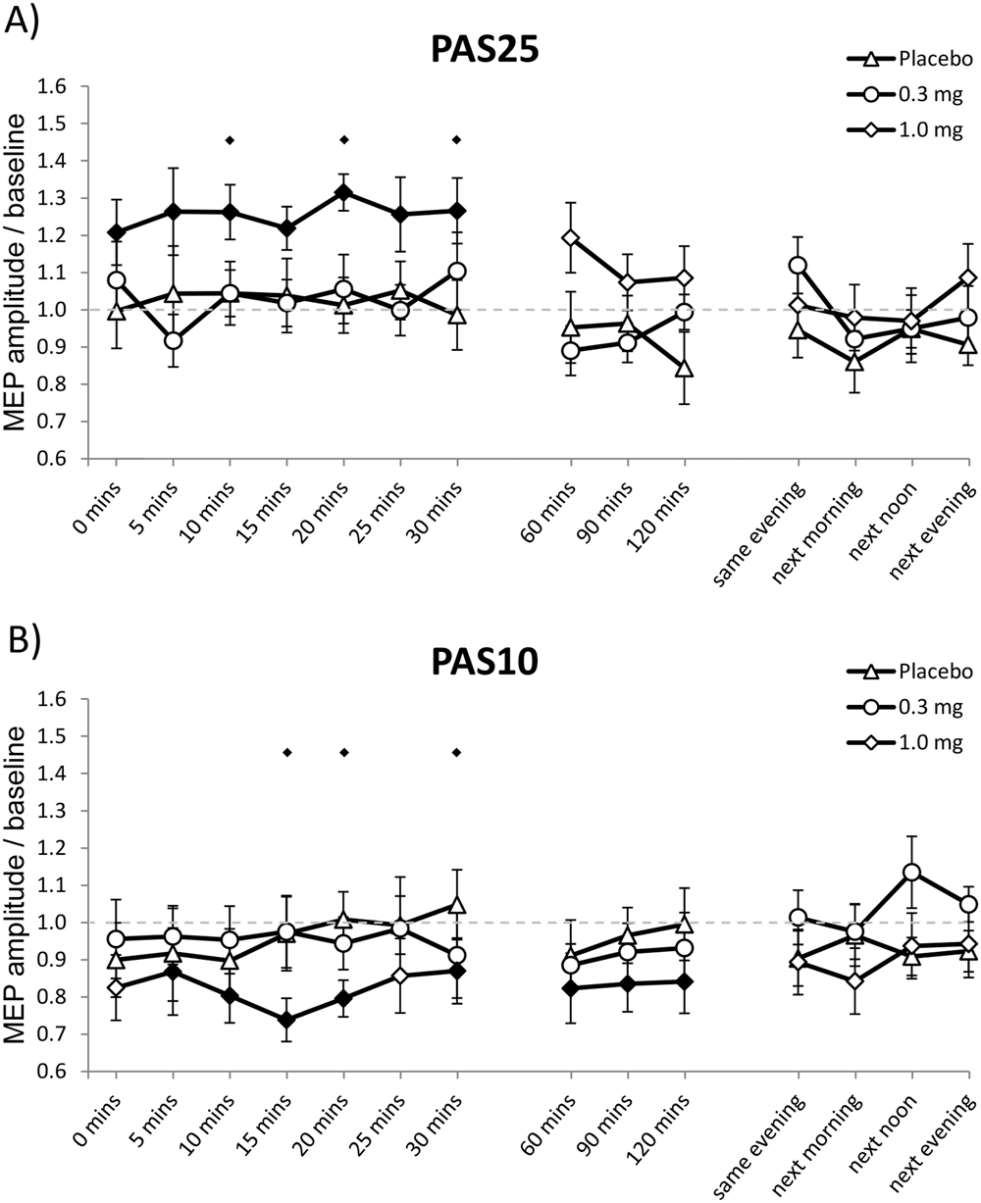
Impact of varenicline on PAS-induced neuroplasticity. Shown are baseline-normalized MEP amplitudes after plasticity induction by PAS25 (**A**) and PAS10 (**B**) under 0.3 mg, 1.0 mg varenicline or placebo medication conditions up to the evening of the post-stimulation day. Both, PAS25 and PAS10 induce no changes of MEP amplitudes in smokers under placebo or 0.3 mg varenicline conditions. (**A**) Cortical excitability was significantly enhanced for up to 30 minutes under high dose varenicline after PAS25 administration. (**B**) High dose varenicline restored inhibitory plasticity after PAS10. Error bars indicate S.E.M. Filled symbols indicate significant differences of post-stimulation MEP amplitudes from respective baseline values; floating symbols indicate significant differences between the respective drug and placebo medication conditions at the same time points (Student’s t-test, two tailed, paired samples, p < 0.05).

Post-hoc Student’s t - tests show that under placebo and low dose varenicline conditions, PAS failed to induce any excitability alterations. Here MEPs did not significantly differ from respective baseline values at any time point. Under high-dose varenicline, MEPs were significantly enhanced for 30 minutes after PAS25 and significantly reduced for 120 minutes after PAS10 (Fig. 4A,B).

For the effects of different dosages of varenicline on PAS-induced plasticity, the one-way ANOVA conducted across groups on the grand average values calculated for the first 30 min after intervention was significant (F(5, 66) = 4.817, p = 0.001).

Low dose varenicline did not restitute PAS-induced plasticity, MEP sizes after both PAS10 and PAS25 were similar to those after in the placebo condition, as revealed by the respective Student’s t-tests (paired samples, two-tailed, p < 0.05). Under high dose varenicline, both PAS25 and PAS10 induced excitability enhancement and diminution respectively, compared to the placebo condition (Student’s t - test, paired samples, two-tailed, p < 0.05) (Fig. 3).

## Discussion

The results of this study demonstrate that nicotine withdrawal and nAChR activation under withdrawal has a notable effect on neuroplasticity in smokers. Under nicotine withdrawal, both tDCS and PAS-induced after effects were abolished, but reinstated by high dose varenicline. High dose varencline also resulted in prolongation of tDCS-induced facilitatory and inhibitory after effects, whereas low dose medication succeeded in restitution of tDCS-induced after-effects, but was ineffective for PAS.

These results are partially similar to those acquired in a previous study, where a nicotine-withdrawal related abolishment of LTP-like facilitatory plasticity was reestablished by global nAChR activation^13^. Thus, we assume that the restitutive effect of nicotine on withdrawal-related impaired plasticity in smoking individuals is in large part caused by activation of α_4_β_2_ nAChRs. Moreover, similarly to another study of our group, conducted in non-smoking individuals, varenicline alone did not produce any effect on cortical excitability^12^.

Non-invasive brain stimulation protocols induce NMDA receptor- and Ca^2+^-dependent LTP- and LTD-like plasticity^40, 41^. Modulation of membrane penetrability to calcium ions impacts upon the induction of LTP and LTD^42^ and thus should be able to alter these after-effects. Nicotinic receptors that influence intracellular Ca^2+^ concentration are α_4_β_2_ and α_7_ nAChRs^43^. These receptors are relevant for LTP and LTD induction^44^, since they influence glutamatergic plasticity through intracellular calcium influx^8, 9^. Thus, administration of nicotine or varenicline leads to an elevated intracellular Ca^2+^ concentration via activation of respective subsets of nAChRs^45^, and affects stimulation-induced after - effects likely through this mechanism^11, 12^.

The lack of LTP-like plasticity in smokers can be explained by desensitization^46, 47^ and/or long-lasting inactivation of nAChRs under withdrawal after chronic nicotine consumption, the latter being observed even up to 5 hours after drug removal in rat brain tissue^48, 49^. Additionally, possibly due to the absence of the stimulatory effect of nicotine, a decrease of glutamate transmission is also observed during nicotine withdrawal^50, 51^. Failure of the facilitatory protocols to induce plasticity under nicotine withdrawal was also observed in a previous study of our group^23^, but here, both inhibitory PAS and cathodal tDCS still induced an excitability diminution. The reason for this discrepancy is unclear, as the groups are similar for age, gender distribution, Fagerstöm scores and withdrawal durations. Possible explanations include inter-group differences of the effects of chronic nicotine exposure on other neuromodulatory systems such as dopamine and serotonin^52, 53^, as well as Brain-derived neurotrophic factor (BDNF) levels^54^, which are shown to have an impact on stimulation-induced plasticity^55–57^. Varenicline administration probably activated α_4_β_2_ nAChRs, which are assumed to be desensitized by chronic nicotine exposure^58, 59^, thus facilitating intracellular calcium influx and therefore enabling either LTP or LTD-like after-effects after tDCS and PAS. Although varenicline is also a full agonist of α_7_ receptors, their impact on the restitutive effect of varenicline on impaired plasticity might be less relevant as (1) its affinity to α_7_ is 4000 to 5000 fold lower as compared to α_4_β_2_^24^, (2) an α_7_ nAChR agonist failed to reduce nicotine withdrawal-associated cognitive deficits, as opposed to an α_4_β_2_ agonist, in another study^60^ and (3) in knockout mice nicotine withdrawal-related deficits in contextual fear conditioning involve β_2_, but not α_7_ subunit-containing nAChRs^61^.

The reason why low dosage varenicline succeeded in restitution of tDCS-induced plasticity, but failed in the PAS experiment can be explained by specific differences between these plasticity-inducing protocols. Since tDCS is assumed to induce plasticity by long-lasting tonic depolarization of relatively large neuronal populations underneath the stimulation electrodes, whereas PAS generates short depolarizations and affects only specific neuronal groups, tDCS might lead to stronger calcium increase, as compared to PAS, for a given individual. A somewhat similar effect was observed in an earlier study of our group, where in non-smoking participants both 0.3 and 1.0 mg varenicline abolished tDCS-induced after-effects, but only 1.0 mg had the same effect in the PAS condition^12^. These results are in agreement with the nicotine-induced calcium overflow in case of tDCS as demonstrated by our recent study^14^, where reduction of calcium influx blocks the conversion of anodal tDCS-induced after effects from excitability enhancement to diminution under nicotine (which is not present for PAS-induced LTP-like plasticity) and thus is compatible with larger calcium influx via tDCS, as compared to PAS.

The abolishment of plasticity in smokers under nicotine abstinence and the restorative impact of varenicline goes in line with respective results of cognitive studies in humans and animals, where α_4_β_2_ receptor-targeting pharmacologic agents ameliorated working memory and attention impairments related to withdrawal after chronic nicotine administration^22, 62^.

The results of this study demonstrate that withdrawal from nicotine leads to deficiency in both LTD- and LTP-like plasticity, but is re-installed after administration of a nAChR agonist which activates receptors with calcium channel properties. These results suggest a key role of α_4_β_2_ nAChRs in pathophysiological alterations under nicotine withdrawal, but also restitution of normal physiological mechanisms. Since nicotine withdrawal also negatively affects such cognitive processes as attention, learning, working memory and response inhibition^21^, results of this study deliver a probable physiological foundation for these cognitive deficits, which however should be explored more directly in future studies.

With regard to the clinical domain, an involvement of the cholinergic system in schizophrenia has repeatedly been suggested^63, 64^. Additionally, the rate of smoking and daily cigarette consumption in schizophrenia patients is significantly higher than in the general population^65^. This led to the assumption that nicotine consumption is a kind of self-medication to improve schizophrenia-related cognitive deficits (“the self-medication hypothesis”)^66^. Recent studies have demonstrated that tDCS and excitatory PAS-induced plasticity are impaired in schizophrenia patients^67–69^. Interestingly, excitability diminishing after-effects of cathodal tDCS are still present in smoking patients as compared to non-smokers^70^. Additionally, non-smoking schizophrenia patients were significantly more responsive to repeated fronto-temporal tDCS sessions, resulting in a decrease in auditory hallucinations compared to smokers^71^, suggesting a complex interaction between impaired plasticity and the cholinergic system. In principal accordance with these studies, demonstrating that neuroplasticity is compromised in schizophrenia, our results can at least partially explain why nicotinic agents may have positive effects in this condition^72, 73^. However, this explanation is hypothetical and should be systematically addressed in future studies.

This study targeted the impact of a single dose varenicline on neuroplasticity during acute (~10 h) withdrawal from nicotine. Plasticity changes related to an extended nicotine withdrawal syndrome, related to smoking cessation, which lasts several weeks^74^, could be significantly different. Additionally, chronic administration of varenicline might lead to discernible neuroplastic and/or cognitive changes as previously reported for nicotine^75^, as well as probable upregulation or desensitization of nicotinic receptors^76, 77^. Therefore, the effect of chronic administration of varenicline on nicotine withdrawal-related plasticity alterations should be addressed in future studies.

It should also be noted that our study explored primarily the impact of α_4_β_2_ receptors on plasticity, however, α_7_ nAChRs have also shown to play an important role for respective processes^44, 78^. For more than a decade, there has been an increased interest to develop high affinity α_7_ nAChR-agonists capable to cross the blood-brain barrier and induce or enhance neuroplastic changes in the brain^79, 80^ for treatment of schizophrenia and Alzheimer’s disease^81^ as well as nicotine addiction^82^. Thus, future studies should be designed to specifically explore the involvement of α_7_ receptors in nicotinic modulation of plasticity in humans.

The subjects recruited for our study were moderate smokers according to the Fagerström scale^26^. As was previously documented, an increase in the intensity of smoking in adults of all ages correlates with a decline in cognitive performance^83–85^, therefore plasticity changes in heavy smokers with stronger nicotine-dependence history may be qualitatively and quantitatively different. A systematic study involving different degrees of nicotine dependency could address this issue. Another limitation is the fact that neither blood nor breath CO tests were conducted to verify compliance of the subjects. Nevertheless, as the experimental sessions were mostly scheduled early in the morning and the participants were moderate smokers, we relied on their statements of compliance. Nicotine abstinence was also not verified after the 120^th^ minute after intervention for the reason that the subjects were no longer present in the laboratory, thus it cannot be excluded that the late (SE-NE time points) neuroplastic effects of the stimulation were affected by nicotine consumption in some participants. Also tobacco craving (For example, Tobacco Craving Questionnaire^86^) and withdrawal (For example, Withdrawal Symptom Checklist^87^), which would have allowed to correlate physiological with behavioral data, was not assessed.

Between baseline 1 and 2 measurements the participants stayed in the local library, therefore it can be safely assumed that they were performing somewhat similar activities (e.g. reading, studying). However, in order to minimize the possible impact of the differences in these activities on the results of the experiment, an interim control would have been advantageous.

Obtaining tDCS and PAS-induced after-effect measures additionally during normal smoking behavior would have added potentially relevant data about plasticity in smokers under naturalistic conditions. This would have however added a substantial amount of experimental sessions, and we decided not to add these assessments, also in light of information we had obtained in a previous study about the impact of controlled nicotine administration in smokers^13^.

Furthermore, our experiment was limited to the motor cortex. Recently, an enhancement of cortical-evoked potentials after prefrontal PAS25 was demonstrated via TMS-EEG^88^. Since cognitive functions that are affected by nicotine abstinence, such as working memory, episodic memory and attention^20^ are related to the prefrontal cortex^89–92^ and can also be modulated by non-invasive brain stimulation protocols^93, 94^ as well as nicotinic agents^1–3^, it would be relevant and feasible to focus the future exploration of nicotine withdrawal-related plasticity shifts directly on prefrontal cortex physiology.

Finally, we did not perform cognitive tests in order to directly connect neurophysiologic results to cognition. Although the results of this study could explain the restitutive effect of varenicline on nicotine withdrawal-related cognitive deficits observed in several studies^22, 62, 95, 96^, this connection is still indirect and remains hypothetical. Thus, future studies should explore the direct relationship between changes in cortical excitability and cognition related to nicotine withdrawal and re-administration.
